# Dataset of wing venation measurements for *Apis mellifera caucasica*, *A. mellifera carnica* and *A. mellifera mellifera* (Hymenoptera: Apidae), their hybrids and backcrosses

**DOI:** 10.3897/BDJ.8.e53724

**Published:** 2020-08-05

**Authors:** Paweł Węgrzynowicz, Aleksandra Łoś

**Affiliations:** 1 Research Institute of Horticulture, Skierniewice, Poland Research Institute of Horticulture Skierniewice Poland; 2 Institute of Nature Conservation, Polish Academy of Sciences, Kraków, Poland Institute of Nature Conservation, Polish Academy of Sciences Kraków Poland

**Keywords:** morphometric analysis, geometric morphometry, honeybee, subspecies

## Abstract

**Background:**

Wing venation is used as a tool in honeybee (*Apis mellifera* L., 1758) subspecies identification. The presented dataset concerns nineteen landmarks located at honeybee worker's forewing vein junctions. Landmarks of *Apis mellifera caucasica* Pollmann, 1889, *A. mellifera carnica* Pollmann, 1879 and *A. mellifera mellifera* Linnaeus, 1758, their hybrids and backcrosses were measured. In total, data from 9590 wings were collected. The dataset could be used in geometric morphometric analysis, studies of degree of inheritance of morphological features and, after further development and supplementation with other local subspecies and hybrids, can contribute to in-depth evolutionary research on honeybees.

**New information:**

Baseline dataset for wing venation of hybrids and backcrosses of *A. mellifera carnica, A. mellifera caucasica* and *A. mellifera mellifera*.

## Introduction

Wing venation is commonly analysed as an important characteristic of insect species (e.g. Owen 2012, Perrard et al. 2014, Rossa et al. 2016, Breitkreuz et al. 2017, Loh et al. 2017, Jacquelin et al. 2018). In honeybees (*Apis mellifera*), the measurement of wing venation is used for subspecies determination (Tofilski 2008, Gerula et al. 2009, Tofilski 2010, Francoy et al. 2012, Santana et al. 2014, da Silva et al. 2015, [Bibr B5553892][Bibr B5553914], Santana et al. 2014, Tofilski 2008, Węgrzynowicz et al. 2019). Due to a significant correlation between morphometric and molecular methods, the measurements and analysis of wing veins are a cost-effective and reliable identification measure (Miguel et al. 2011, Oleksa and Tofilski 2015, [Bibr B5889338][Bibr B5744242]). Geometric morphometrics, based upon nineteen landmarks located at honeybee worker's forewing vein junctions, has been used in Poland since 2008 as a tool for assessing subspecies identity (Tofilski 2008, Gerula et al. 2009). Creating databases that not only include representatives of various subspecies, but also data from hybrids and backcrosses, seems to be particularly valuable. This Data Paper provides raw metadata freely available for further exploration, which can be correlated, for example, with the reference samples for the subspecies from the Morphometric Bee Data Bank in Oberursel, Germany. It may also be used to assess inheritance of morphological features.

## Sampling methods

### Sampling description

The original colonies of subspecies came from long-term breeding. *Apis mellifera carnica* and *A. mellifera caucasica* come from colonies maintained within a selection carried out by the Research Institute of Horticulture. *A. mellifera mellifera* was supplied from a conservation breeding programme supervised by the National Animal Husbandry Center (https://www.kchz.agro.pl/wykaz-ksiegi-1_03_2019_1/). These subspecies are commonly maintained in Poland. Methodology described by Węgrzynowicz et al. (2019) was used in order to obtain the parental generation of bees from *A. mellifera carnica, A. mellifera caucasica* and *A. mellifera mellifera* subspecies, their hybrids and backcrosses. Two hundred queens with 24 different genotypes have been obtained, representing all possible hybrid and backcrosses combinations of *Apis mellifera carnica*, *A. mellifera caucasica* and *A. mellifera mellifera* subspecies. To conduct morphometric analyses, worker bee samples were taken shortly after emergence directly from incubated combs. Bees were stored in plastic containers filled with 96% ethyl alcohol at room temperature for 4 to 5 months. After this period, each worker bee had its right wing removed. The wings were mounted in a glass photographic frame and scanned with a Nicon Coolscan 500 ED scanner (image resolution 2400 dpi, greyscale). Wing images were analysed with DrawWing software (freely available at: http://drawwing.org/node/2) to determine the coordinates of nineteen landmark features (Suppl. material 1). The dataset (Suppl. material 2, Suppl. material 3) contains parameter measurements for individually analysed honeybee wings.

## Geographic coverage

### Description

The Institute of Horticulture, Apiculture Division in Puławy, Poland. Altitude above sea level: 137 m.

### Coordinates

 and 51.246 Latitude Latitude; and 21.581 Longitude Longitude.

## Taxonomic coverage

### Description

Wing venation is a suite of characteristics that allows the identification of subspecies. Locally-sustained bees of *A. m. caucasica*, *A. m. carnica* and *A. m. mellifera* with stabilised wing parameters were used to create hybrid and backcrossed workers and all were included in our baseline dataset.

## Temporal coverage

**Data range:** 2013-5-20 – 2015-9-15.

### Notes

Beekeeping seasons in Poland.

## Usage rights

### Use license

Creative Commons Public Domain Waiver (CC-Zero)

## Data resources

### Data package title

Dataset of wing venation measurements

### Number of data sets

2

### Data set 1.

#### Data set name

Wing_venation_dataset

#### Number of columns

7

#### 

**Data set 1.** 

Column label	Column description
No.	serial number
Queen/Colony ID	Queen/Colony ID
Maternal origin ID	Maternal origin ID
Fatherly origin ID	Paternalorigin ID
GENOTYPE	GENOTYPE - short name which is described in the "Genotype_description" set
WINGS ID	WINGS ID - individual ID given to each sample from a combination of: "No.", "Queen/Colony ID", "Maternal origin ID", "Fatherly origin ID", "GENOTYPE"
x0, y0 - x18, y18	Parameter coordinates (in separate columns)

### Data set 2.

#### Data set name

Genotype_description

#### Number of columns

4

#### 

**Data set 2.** 

Column label	Column description
GENOTYPE	GENOTYPE
Number of measured wings	Number of measured wings
Colonies amount	Colonies amount
Abbreviation description	Abbreviation description [P0, P1 - parental generations, F1 - hybrid generation, BC - backcrosses generation; lowercase letters represent the subspecies: first letter: parental-queen, second letter: paternal-drone (m - *A. m. mellifera*, k - *A. m. carnica*, c - *A. m. caucasica*)]

## Supplementary Material

8FCEC327-C973-5C17-A18D-C1DDE1AE98F1 10.3897/BDJ.8.e53724.suppl1 Supplementary material 1 Image of the x and y coordinates obtained with the DrawWing sofware for 19 vein connections on the right wing of worker honey bee Data type Image File: oo_422984.tif https://binary.pensoft.net/file/422984 Paweł Węgrzynowicz

CF5B3D52-DC57-5BC0-A3C3-C39E2FEEA43F 10.3897/BDJ.8.e53724.suppl2 Supplementary material 2 Dataset of wing venation measurements - abbreviations descriptions Data type txt Brief description Descriptions of the abbreviated names of the genotypes used in the dataset of wing venation measurements. File: oo_404831.txt https://binary.pensoft.net/file/404831 Paweł Węgrzynowicz

B37C5416-12E6-5689-85CA-92D459BBA7A1 10.3897/BDJ.8.e53724.suppl3 Supplementary material 3 Dataset of wing venation measurements Data type txt Brief description The database contains the x and y coordinates obtained with DrawWing sofware for 19 vein junctions on the right wing of worker honeybees (38 coordinates for each wing). The analysed wings originate from offspring of 200 queens with 24 different genotypes representing all possible hybrid and backcrosses combinations of *Apis mellifera carnica*, *A. mellifera caucasica* and *A. mellifera mellifera* subspecies. Results from a total of 9590 wings are presented in the dataset. File: oo_404830.txt https://binary.pensoft.net/file/404830 Paweł Węgrzynowicz
