# Supplementary figures and images for: Dataset of wing venation measurements for Apis mellifera caucasica, A. mellifera carnica and A. mellifera mellifera (Hymenoptera: Apidae), their hybrids and backcrosses
Source: Biodivers Data J. 2020 Aug 5;8:e53724. doi: 10.3897/BDJ.8.e53724 (PMC7423776; doi:10.3897/BDJ.8.e53724)

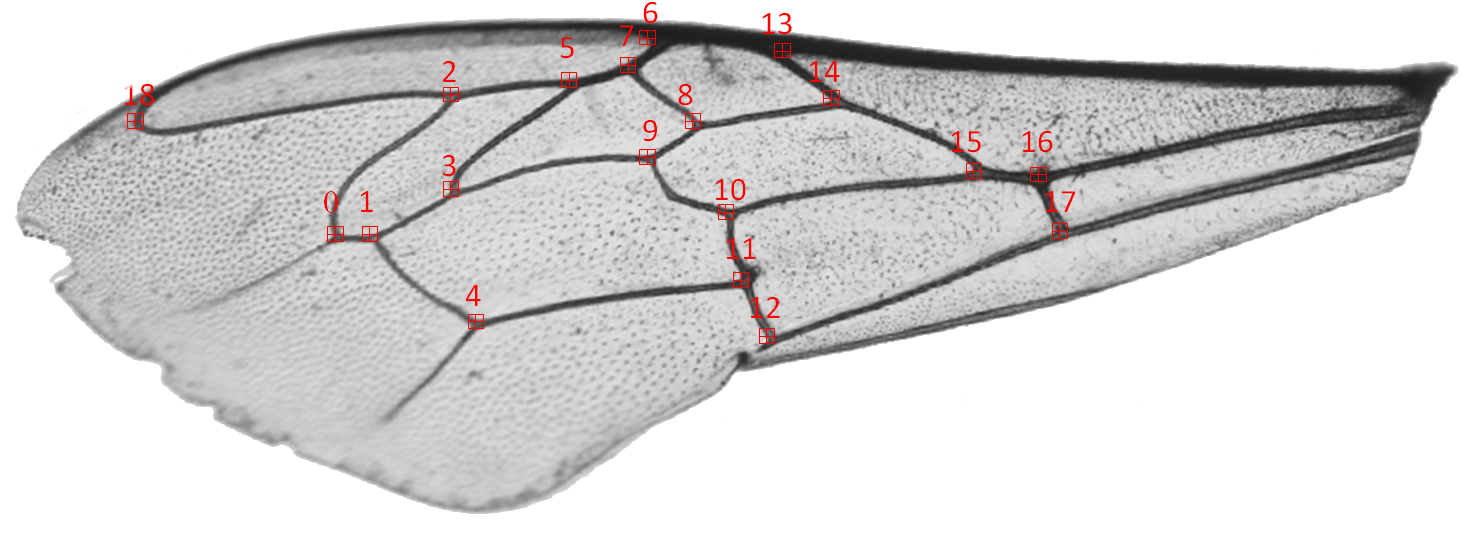

Supplement: Supplementary material 1 — Image of the x and y coordinates obtained with the DrawWing sofware for 19 vein connections on the right wing of worker honey bee [file bdj-08-e53724-s001.tif]
